# The social representations of Covid-19 among primary health care’ users in the Federal district, Brazil: A psychosocial approach

**DOI:** 10.1371/journal.pone.0323568

**Published:** 2025-05-27

**Authors:** Helena Eri Shimizu, Antonio Marcos Tosoli Gomes, Thémis Apostolidis

**Affiliations:** 1 Department of Collective Health of Faculty of Health Sciences of University of Brasilia, Brasília, Brazil; 2 Department of Nursing Medical Surgical of UERJ: Universidade do Estado de Rio de Janeiro, Rio de Janeiro, Brazil; 3 Aix Marseille University, LPS, Aix-en-Provence, France; Sergio Arouca National School of Public Health: Escola Nacional de Saude Publica, BRAZIL

## Abstract

Brazil was one of the countries most affected by the COVID-19 pandemic, including severe psycho-social effects. This study aimed to analyze the Federal District Primary Health Care users’ Social Representations (SR) regarding Covid-19 through exploration of the elements that comprise their field, identification of the variations of their prominence, as well as analyze the relations and differences among them according to their socio-demographic characteristics and the participants’ exposure to the disease. It was based on the Moscovici theory of social representations. 1,714 users from the Federal District Primary Health Units regions participated in the study: Central-South, North, West, Southwest, East and South. For the data collection, the free evocation technique was used. The data were analyzed by the Iramuteq software in the prototypical modality, analyzing similitude and analyzing x^2^ test of the most prominent words, considering the socio-demographic and Covid-19 exposure variables, establishing p < 0.05. It has been found that the social representations of the pandemic among the Federal District PHC’ users formed a dyad: doctor and death. It was observed, however, that the doctor-death relationship was organized according to the vulnerabilities present in the face of the pandemic context and the affective-emotional aspects, the most excluded social segments experiencing the terrifying feelings caused by the virus, particularly the confrontation with death and its consequences, along with the absence of hope. In particular, it was evident that the representation of an absent or omissive State, due to the lack of protective measures for people already socially excluded, who demanded agile, more equitable policies, configured from the perspective of bioethics a moral debt on the part of the State to this population.

## Introduction

Brazil was one of the countries most affected by the Covid-19 pandemic, the mortality rate from January 2020 to February 2021 having reached 119 per 100,000 [1]. The Federal District (FD) was the first state/municipality to decree a lock down [2], and possessed good infrastructure, including the number of hospital beds, at the time, situating it as one of the only two state capitals, the other being Rio de Janeiro, which had better conditions to provide proper care [3,4].

However, after about a month, the FD, as occurred in most Brazilian cities, began to introduce services, in principle, considered essential. This movement was not accompanied by proper testing of the population, exposing everyone to contact with the virus, resulting in a rise to 155 per 100 thousand from January 2020 to February 2021 [4].

Given this prolonged scenario, the population was exposed to feelings of general panic, since they faced a little-known disease that put them into social isolation, generating much anguish. In this respect, it is important to mention that, due to the contagion of the virus and the social distancing mechanisms implemented, the relationship with others often began to constitute a situation of confrontation, at risk of contracting and spreading the virus [5].

The construction of the object, disease as an essential part of social norms and relations activates paths of thought, not only contained in the object itself (in the ontological sense), but also in the subject that represents it (in the epistemic sense) [5,6]. From this perspective, Covid-19 may be a powerful revealer of individual and collective realities, confronted with a sudden rupture of social normality. The formation of Social Representation (SR) is crossed by cognitive and affective dimensions, with an important role played by the emotional context in individual and collective symbolic thinking [7,8,9,10,11], which find their expression in the words uttered about Covid-19, ones that need to be recognized and understood better [11,12].

In addition, it is observed that, background behavior in the face of Covid-19, social norms and values, symptomatic of inequalities, positions and social relations may be contained. The pandemic can reveal exclusion/marginalization, above all from some social groups, so that socio-medical perceptions constituted by xenophobia, stigma and prejudice function as strategy to blame the victims [12]. Furthermore, a reading of the inequalities and social and psycho-emotional vulnerabilities caused by Covid-19 [13] is necessary, taking into account the theoretical-political reference of intersectionality and social bioethics [14], which allows analysis, in greater depth, of particular historical contexts, situational power relations, the structural processes of oppression to which they were subjected [15].

In this sense, it is necessary to listen to the Primary Care’ users who experienced the daily processes resulting from the pandemic, in a context that showed potency in facing the disease, especially due to the recognition that individual hospital care was insufficient [16]. Primary Care (PHC) users were chosen as participants in this study because it is the main entry point, except in cases of extreme emergency, and also from where they are referred to other services, when necessary [16]. This study is justified by considering that it is a phenomenon that goes beyond the biological dimension and is strongly manifested in the social dimension, in which strong, integral PHC contributes at full capacity.

The study aimed to analyze the social representations of SUS Primary Care’ users around the time of the pandemic, through exploration of the elements of the fields of these representations, identification of the variations of the more prominent elements as a function of the socio-demographic characteristics and health of the participants, as well as the analysis of the relations and differences among the most prominent elements as a function of these same variables.

## Method

The Social Representations approach has proved proficient for studying that pandemic phenomenon, crisscrossed by tension zones with experiences of turmoil in everyday life, which sometimes brought into play the legitimacy of scientific and secular knowledge. Moreover, Social Representations (SR) theory enables understanding of social thinking from human relations, collectively shared and that aid daily conduct and practices [5,6].

It was an exploratory study, conducted in the Federal Capital District of Brazil, which combines State and municipal functions, when to population was 2,817,381 [17]. There is a regionalized public health system, subdivided into seven Health Regions (RAS), in which PHC is the main gateway for the user and the co-ordination of the system.

The participants in the study were users of the Basic Health Units (UBS/PHC) located in the Central-South. North, West, Southwest, East and South regions, except Central, who obeyed the following user inclusion criteria: aged over 18, registered with the Family Health Strategy, main PHC services, at least six months ago. And those of exclusion were: did not present physical conditions, did not communicate verbally, and were not oriented in time and space to answer the questionnaires. The sample was based on the number of users of each UBS/PHC from the six RAS, whose population was 607,695, obtaining 1,714 people, considering 20% losses. Therefore, data collection in multiple regions aimed to improve data homogeneity.

### Inclusivity in global research

Additional information regarding the ethical, cultural, and scientific considerations specific to inclusivity in global research is included in the Supporting Information (SX Checklist).

### Data collection and analysis

A questionnaire was applied to UBS/PHC users, containing questions with the following socio-demographic variables: gender, race/color, marital status, education, income (considering a median value of US$ 370), occupation, whether or not a social benefit of around US$ 120 per month was received (federal or State government), covered by health insurance or not. Then the free evocation technique was applied [18], in which users were asked to say three to five words that came to mind when the word Covid-19 was heard. At the end, the user was asked if he/she had had the disease, and equally whether the family had had it. Data collection occurred in person in UBS/PHC units from 01 June 2021–20 December 2022, when the researchers and other users were receiving the first vaccine dose, followed by the equity criteria: elderly, “quilombolas” (descendants of fugitive slaves), and indigenous people.

### Data analysis

The textual corpus was analyzed with the aid of Iramuteq software ((Interface de R pour les Analyses Multidimensionnelles de Textes et de Questionnaires), version 0.7 alpha 2. The software utilizes an R interface (www.r-project.org) that enables textual data analysis procedures based on lexicometry, and realizes calculation of multivariate frequencies and analyses of words [19].

The first procedure performed was prototypical analysis of the data with the purpose of exploring the most prominent cognitive content produced through the verbal association technique [20,21]. This technique allows identification of the level of prominence of the content, based on the intersection of two criteria: the frequency of appearance of the word and mean order of the appearance of the evocations [22]. In other words, these criteria complement each other, in addition to providing two collective indicators to characterize the prominence of a word in a corpus generated from a social group. These results can be visualized in a contingency table with four quadrants. In the first quadrant (upper left) and the second (upper right), there are the most prominent evocations. In the third (lower left and right) there are the evocations of lower prominence.

The second procedure performed, with all the words that had arisen in the first and second quadrants of the prototypical analysis, was analysis of the variation of the presence of these words, through the x2 test of the words, establishing p < 0.05, considering the socio-demographic variables: gender, race/color, marital status, education, income, occupation, social security beneficiary or not, health insurance or not, and health in relation to Covid-19, whether the user and/or family member(s) had had it or not. This procedure was intended to observe intragroup differences in the representations of Covid-19.

The third was similtude analysis [23,24] of the ten most prominent words to explore the structure of the internal organization of the Covid-19 social representations. This analysis is based on the connectivity criterion that allows exploration of the statistical force between two representational elements, based on the Jaccard index that presents a statistic between 0 (absence of relation) and 1 (strong relation), besides allowing the possibility of visual observation of word communities [25].

The fourth and last performed similar analysis, choosing three socio-demographic and health variables of the ten most prominent words, aiming to understand the connections of the psycho-social and emotional aspects of Covid-19 exposure.

### Ethical considerations

The research project was approved by the Research Ethics Committee of the Health Sciences Faculty of Universidade de Brasília (UnB), registered under number 4.413.580. All participants signed in written form the Free Informed Consent Term.

## Results

1,714 questionnaires were answered, containing 4,829 words and 488 distinct words. As for the respondents’ profiles, most PHC users (Table 1) were female (81.51%), brown (63.41%), without a partner (56.30%), with a high school education (54.78%), income of up to US$ 370 or 2 thousand Brazilian reals (50.40%), and with formal employment (57.99%). Among them, 77% did not receive a social security benefit, and 88.85% had no health insurance. Besides this, it was observed that 67.67% had not had Covid-19, and neither had 64.76% of families.

**Table 1:** Profile of respondents/ users of the Federal District PHC services.

Gender	Frequency	Percentage
Female	1,397	81.51
Male	317	18.49
**Race/Color**		
White	361	21.06
Brown	1,087	63.42
Black	224	13.07
Other	42	2.45
**Marital status**		
With partner	749	43.70
Single	965	56.30
**Education**		
Primary complete and incomplete	295	17.21
High school complete and incomplete	939	54.78
Further complete and incomplete	480	28.00
No education	9	0.5
**Income**		
Less than	864	50.40
Over	466	27.18
Refused to declare or did not know how to respond	384	22.40
**Occupation**		
Formal	994	57.99
Informal	333	19.42
Unemployed	374	21.82
**Social Security**		
Benefit	393	22.92
No Benefit	1,321	77.07
**Health Insurance**		
Insurance	191	11.14
No Insurance	1,523	88.85
**Covid-19**		
Had it (C)	554	32.32
Did not have it (NC)	1,160	67.67
**Covid-19 in the Family**		
Had it (HCF) (FCC)	604	35.23
Did not have it (NCF) (FNC)	1,110	64.76

The findings demonstrated in Table 2 reveal that, situated in the first quadrant, are the elements with the highest prominence that comprise the SR, with an average evocation order (EMO) of <2.22 and frequency 63, and those of the first periphery >2.22 and frequency 72. The words: doctor and death are highlighted in the prominence of the Covid-19 SR. The words: sadness, anguish, disease, fear and hope portray the feelings experienced by people at the height of the pandemic. The words: virus, despair and evil refer to the search for explanations for the events at that time.

**Table 2:** Prototypic analysis regarding the inductor term, Covid-19.

Central Nucleus			First Periphery		
Evocation mean order < 2.22			Evocation mean order > 2,22		
Word	Freq	EMO*	Word	Freq	EMO*
Doctor	549	1.5	Isolation	173	2.9
Death	459	1.9	Loss	132	2.7
Sadness	207	2.1	Family	101	3.1
Anguish	141	2.1	Care	94	2.5
Disease	118	2.1	Anxiety	94	2.9
Fear	116	1.6	Vaccine	84	2.8
Hope	101	1.9	Insecurity	72	2.5
Pandemic	84	2.0			
Virus	77	2.1			
Despair	36	2.0			
Evil	63	2.1			
**Contrast Zone**			**Second Periphery**		
Affliction	52	1.6	Health	53	2.8
Change	37	2.2	Worry	43	2.6
Dread	35	2.1	Contamination	38	2.5
Suffering	35	1.9	Distancing	35	2.9
Horrible	33	1.6	Panic	34	2.4
Missing	28	2.2	Unemployment	35	2.5
Learning	26	2.2	Loneliness	3.1	2.7
Terror	26	1.6	Routine	29	3.1
Chaos	18	2.1	Mask	28	3.1
Danger	17	2.1	Persons	28	2.8
Destruction	15	1.8	Depression	27	3.3
Frightening	14	1.6	Difficulty	24	2.3
Pain	12	1.8	Mourning	23	3.0
Tragedy	12	1.8	Prevention	23	2.7
Plague	11	1.4	Stress	20	2.3
			Life	19	2.8
			Social	17	3.1
			Psychological	17	3.9
			Respect	17	3.1
			Friends	15	3.9
			Sequels	14	3.3
			Dissatisfaction	14	2.7
			Hygiene	13	3.0
			Alcohol	13	2.8
			Disregard	13	2.8
			Uncertainty	12	2.5
			Hunger	12	2.4
			SUS	12	2.8
			Information	12	2.9
			Love	12	3.5

*EMO = Evocation mean order

On the other hand, in the second quadrant, the words: isolation, loss, family, portray the direct effects of the pandemic, especially people in closest contact. Moreover, there were words that were evidence of current feelings and necessary care, but the anxiety was great, since there was not sufficient vaccine for all, given that they were still considered unsafe and would not ensure everyone’s life at that time. The third quadrant portrayed the feelings arising from the changes and sufferings caused by the pandemic. The fourth showed the State/government’s negligence, which caused the lack of care provided by SUS,

In this study we worked with the most prominent elements, those considered socially more important being presented in the first and second quadrants.

Table 3 shows the results of the x2 word test according to the variables. For the gender variable, it was observed that the words fear and hope were significant, and more so among men than women. Regarding race/color, it was found that the presence of the word disease was greater for blacks than for brown and white people. The word hope was more common among whites than for the black and brown population. Losses were more important for brown people than for whites and blacks. As for marital status, it was found that people without partners emphasized the words death and hope more than those with a partners. Also, the words loss and family were more prominent among people with a partner than those without.

**Table 3:** Analysis of the x^2^ test of the most frequent words, considering the socio-demographic and exposure variables for Covid-19.

	Gender	Race/Color	Marital Status	Income	Education	Social Security Benefit	Health Insurance	Covid-19	Covid-19 in the Family
	Male (M) Female (F)	Black (BL) Brown (BR) White (W)	Single (S) With Partner (WP)	Less than US$370 or 2 thousand Brazilian reals (I<) Over US$370 or 2 thousand Brazilian reals (I>)	Primary school (P) High school (H) Further School (F)	With Benefit (B) No Benefit (NB)	With Insurance (I) No Insurance (NI)	Have Covid-19 (C) No Covid-19 (NC)	Family with Covid-19 (FC) Family not have Covid-19 (FNC)
Prominent words									
Doctor				(I<)>(I>)*	H > P,F*		NI > I*		
Death			S > WP*	(I>)> (I<)*	P > F,H*	NB > B*		NC > C*	FC > FNC*
Sadness					P > F,H*				
Disease		BL > BR,W*							
Fear	M > F*			(I>)>(I<)*	F > H,P*		I > NI*		
Hope	M > F*	W > BR,BL*	WP > S*		F > H,P*	NB > B*		C > NC*	
Evil					P > H,F*	B > NB*			
Anguish								C > NC*	
Pandemic								NC > C*	
Virus								NC > C*	
Despair									FC > FNC*
Loss		BR > W,BL*	WP > S*		P > F,H*	B > NB*	NI > I*		
Family			WP > S*		P > H,F*				
Care								NC > C*	
Anxiety								C > NC*	
Vaccine							NI > I*		

*p < 0.05

Regarding the term income, it was found that those with low earnings evoked the word more than those with higher levels. The words death and fear were more prominent among people with the higher incomes than those with the lower ones. Regarding education, the word doctor was uttered more by people with high school level than those with primary and further levels. Among people with primary schooling the words death, sadness, loss, badness and family were more frequent than among those with high school and further levels.

Regarding social security benefits, the words death and hope were highlighted more among people without benefit than those who received one. The presence of the words badness and loss was greater in people with a benefit than without it. Regarding having health insurance, it was identified that people without exalted the words doctor, loss and vaccine than people with. Furthermore, people with health insurance highlighted the word fear more than those without.

As for having had Covid-19, it was observed that the words death, pandemic, virus and care were more prominent among people who had not had it than those who had. Moreover, the words, hope, anguish and anxiety were more common among people who had had it. Users with Covid-19 in the family highlighted the words death and despair more than those who had not had it.

**Fig 1:**
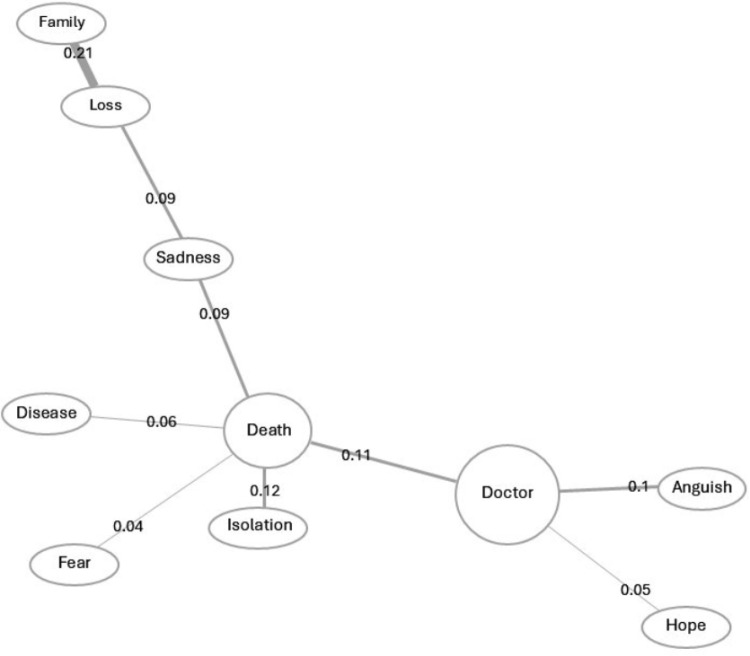
Analysis of general similitude.

Similitude analysis, conducted with the ten most prominent words (Fig 1), shows that the representation is organized around two lexical groups: death and doctor. Moreover, it shows that, around the word death, others are agglutinated: isolation, disease and fear. Death, in this structure, presents the largest number of connections (5), while doctor has three. In turn, doctor is linked, beyond death itself, with anguish and hope. On the other hand, sadness is linked to loss and family. Analysis of the degree of binding force between words, loss and family (0.21), death and isolation (0.12), doctor and anguish (0.1), and doctor and hope (0.05) are outstanding.

**Fig 2:**
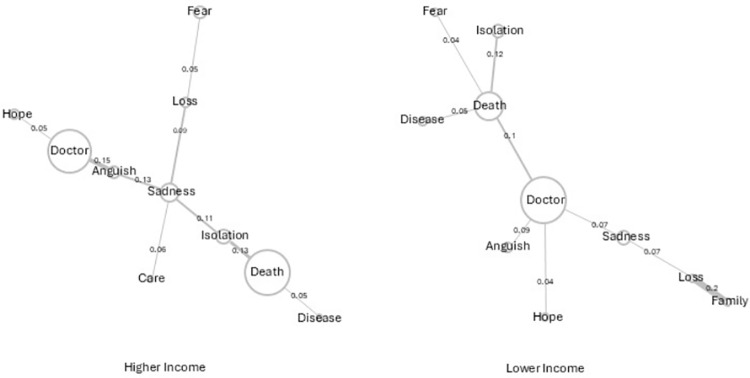
Similitude analyses of users with incomes greater or less than (US$ 370) or 2,000 Brazilian reals.

In the similitude analysis represented in Fig 2 - Users with higher and lower incomes - it was observed that there were differences in the meanings of the words that comprise the representation. In higher income users, the relationship between the words doctor and death is mediated by the word sadness, which is linked to the first through anguish, and the second through the word isolation. There is also the specific presence of the word care. In lower income users, the relationship between the doctor and death words occur directly. The word death has a strong link with isolation (0.12), disease and fear.

**Fig 3:**
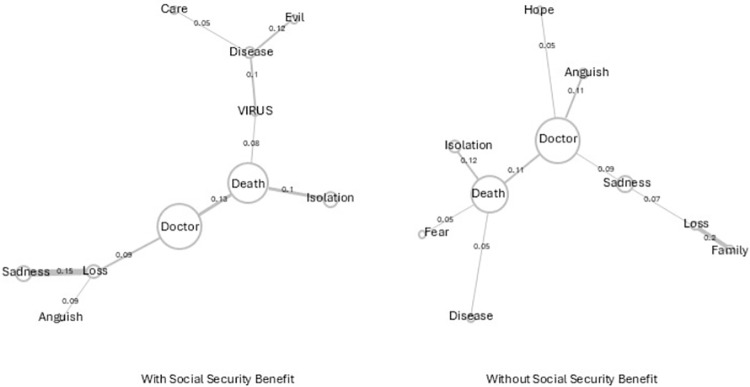
Similitude analyses of users with and without social security benefit.

Fig 3 - Users with and without social security benefit - shows that the words doctor and death, for both groups, have a direct connection. In addition, differences are shown in the presence of the words, with emphasis on evil and care in the group that receives the benefit, and fear and hope in people who do not.

**Fig 4:**
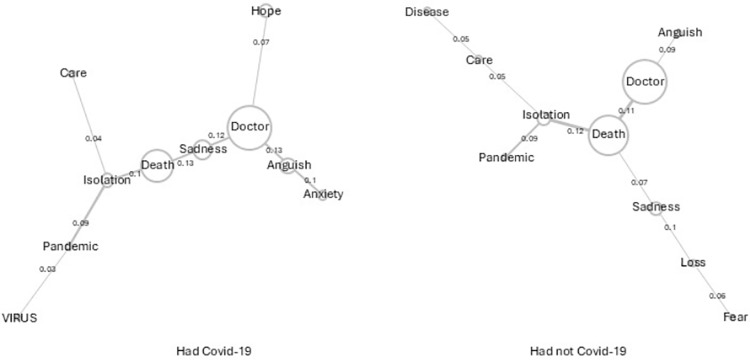
Similitude analysis of users who had had Covid-19 and those who had not.

In Fig 4 - Users with and without Covid-19, it appears that, in the tree of similitude of those who had had Covid-19, the words doctor and death are connected by the word sadness. Also, the exclusive words of this tree are hope, anxiety and virus. In the similitude tree of people who had not had Covid-19, the relationship of the word doctor and death is direct. Besides this, a highlighted presence of the words: disease, loss and fear is observed.

## Discussion

In this study, it was found that the PHC users’ social representations of Covid-19 are organized around the dyad: doctor and death, because in the prototypical analysis, these were the words that were most frequently evoked, 549 and 459 times, and are among the lowest mean rank of the order of appearance, 1.5 and 1.9, respectively. These results indicate the salience of these contents in the SR. Observing the general similarity analysis, they are the elements with the greatest number of connections—death with five and doctor with three—indicating their significant role in structuring the representation. Moreover, it was observed that there were similarities and differences in the forms of psycho-emotional expression among these users, resulting from modes of facing the pandemic [10,11] that involved a long period of social isolation.

The social representation of Covid-19 (and its most mortal expression) is anchored in the severity of the disease that assailed the country, with 892,953 Covid-19 cases registered in the FD, including 11,838 deaths by 2022 [26]. The pandemic changed the rites of death and passage [27], did not allow farewells due to the highly contagious nature of the virus, co-operating to produce extremely negative psychological results, prolonged for individuals and family members [5,12]. Such social representations have shown that some common feelings are shared in the face of this social crisis.

It is noteworthy that, from the analysis of the empirical data, it was possible to identify the elements that comprise the representational field, as well as the variations of these same elements and to understand the internal relations established as a function of the socio-demographic and health variables. For instance, the elements ‘death’ and ‘loss’ have the highest number of vulnerability variables. ‘Death’ is associated with participants without a partner, with primary education, without social benefits, and with family members who had Covid-19, while ‘loss’ is associated with individuals of mixed race, with primary education, and without health insurance. These analyses allowed perception that the representations of Covid-19 were organized around the vulnerabilities present in the face of the pandemic context, and the affective-emotional aspects, the result of group engagement and the participants’ individual and collective behavior.

The most vulnerable, here understood as psycho-social in character, who also include those with individual susceptibilities [28], presented, in a specific manner, more pragmatic elements in the structure of their representations, such as disease (more associated with blacks), doctor (more associated with those with lower income and no health insurance) and vaccine, as well as for those without a heatlh insurance. Regarding the psycho-social dimension of affection, there is an important difference, since those who are considered most vulnerable emphasized sadness (those who have only a primary school education), while those not or less vulnerable pointed out fear (men, those with higher incomes and those with health insurance). The fear of Covid-19, with a strong presence in this group, particularly for those who had had Covid-19, as well as their families, conceals, under this label, a wide variety of emotions, such as feelings of anguish, insecurity, despair and anxiety [10,11]. On the other hand, fear, when it is not paralyzing, can trigger positive feelings, with emphasis on hope for this group, which has provided them the possibility of a future living without Covid-19 [10].

In this study, we opted to understand social vulnerability from the perspective of critical bioethics, since it helps unravel some specific forms of exclusion that reinforce human fragility, and are not related to purely socio-economic conditions [14,28], nor to the mode of organization of State policy, in which there can be non-egalitarian treatment, which exposes the subjects to different forms of vulnerability, originating from the social and quotidian conditioners of their lives [28].

The vaccine arrived in the country in earnest, above all due to problems of poor political governance [29], only in January 2021, which, at first, required scaling the established priorities considering the equity criterion, an important aspect highlighted by Intervention Bioethics [14] and Empirical Bioethics [30] as a form of protection of the most needy during the pandemic. At the beginning of the vaccination process, in the PHC UBS there were many long queues and for a long time [31], but the entire population had the right to receive it for free through SUS, the country’s universal public health system.

It was also found that the social representations of Covid-19 were permeated by feelings of guilt, which stimulated adaptive mechanisms seeking survival and answers [10,11]. In this context, the most vulnerable PHC users, those with only a primary school education and received a social security benefit, revealed that their lack of knowledge about the origin of the disease led them to represent it as evil (in the words of the participants), as punishment. This process occurred in the face of the need to anchor it, as Moscovici explained, that is, to make this new unknown object, the Covid-19 virus, familiar [5,6,32]. Other new diseases that caused fear and panic also led to similar mechanisms, such as HIV, ebola, H1N1, similar to what happened in the past with Spanish flu [5,11].

Other studies have shown that the most vulnerable social groups, who were already excluded previously, were those who had to expose themselves to protect the lives of others, that is, the service staff considered essential [33]. Therefore, death for them was more of a threat [33], and they had less hope, especially the black and brown population [15]. From the perspective of intersectionality and bioethics, the lack of proper protection of these people during the pandemic is characterized as a serious moral failure [34].

Another risk exposure factor for these most vulnerable people was the lack of conditions for distancing/isolation, since they lived in homes with small rooms making contamination more likely [35]. This occurred mainly on the outskirts of the biggest cities and more needy districts, including in the FD, which also presents great disparities of standards of living [36]. In these places people were already isolated from mainstream urban life [36] due to lack of investment in sanitation, paved streets, public transport, and facilities that comprise the social protection network.

In addition to the sanitary crisis, another consequence of the pandemic experienced by this social group was unemployment and deregulation of labor [37], which resulted in reduction or lack of income [33], leading the group to resort to aid of a humanitarian nature to survive. This population was not adequately assisted with policies aimed at protecting it from hunger and poverty, especially in view of the delay in the implementation of this emergency aid as well as its low value, not allowing even minimal subsistence conditions [33,37]. This is characterized as people subjected to moral vulnerability or excluded and socially stigmatized [28].

It was observed that, from the point of view of bioethics, the exposure to Covid-19 was characterized as a contingent, socially produced situation, which contributed greatly to the exclusion or marginalization of some more vulnerable population groups: populations that have incapacities that preclude them from facing their own helplessness, as they need protection to face adversities [38–40], as well as deprivation from access to social and technological benefits, which would justify more protective action on the part of the State [34,38,39].

Besides this, there was a deepening of social inequalities, which was exacerbated due to the adoption of a case mitigation policy, including in the FD. The feeling of abandonment by the State was evident, particularly among the socially excluded, who were more exposed to all the risks [33,34,35], this constituting a serious moral debt [34].

The limitations of this study refer to the method, because the free evocation technique only allows identification of sharing with regard to representation elements through more evident quantitative intragroup consensus patterns [21,22], although analyses of the meanings of the words were performed to understand their differences in the group, as well as similitude analyses to comprehend the internal organization of the SR. Another limitation was the high percentage (more than 80%) of participants who did not have Covid-19 nor experienced it among family members; however, the fact that 20% had the experience of dealing with the consequences of a highly devastating new disease may reveal significant psychosocial aspects. It is worth noting all the difficulties related to the data collection in the context of the pandemic, which required researchers to take great care that is, wearing the necessary protective attire: aprons and masks, cleansing with alcohol gel, as well as receiving the vaccines. On the other hand, it was a moment of important interaction with users, mostly low-income women, who were very apprehensive, so it was also a moment for clarification of doubts about Covid-19. The study of SR was heuristic to understand the social vulnerabilities to which PHC users were exposed in the context of the pandemic. The acute phase of the Covid-19 pandemic has passed; however, it left a legacy highlighting the need to better prepare health systems, especially PHC, by strengthening actions involving psychosocial care for users, as well as health communication aimed at preventing and promoting physical and mental health.

## Conclusion

It is concluded that the social representations of the Covid-19 pandemic of the FD PHC users are most organized around the dyad: doctor and death. It was observed, however, that the doctor-death relation was organized around the vulnerabilities present in the face of the pandemic context and the affective-emotional aspects, and the most excluded social segments experienced the terrifying feelings caused by the virus, particularly the confrontation with the prospect of death and its consequences, and absence of hope. In particular, it was evident there was representation of an absent or omissive State, due to the lack of protective measures for people who were already socially excluded, required agile, more equitable policies, thus configured, from the perspective of bioethics, as a moral debt to this population on the part of the State.
